# Risk factors for bronchiolitis obliterans complicating adenovirus pneumonia in children: a meta-analysis

**DOI:** 10.3389/fped.2024.1361850

**Published:** 2024-08-01

**Authors:** Mei-mei Yao, Tian-ji Gao, Min Zhao, Yan-hua Fu, Jing Liu, Tian-jiao Wang, Ying Yang

**Affiliations:** Department of Rheumatology and Immunology, Baoding Hospital, Beijing Children's Hospital Affiliated to Capital Medical University, Baoding, China

**Keywords:** children, adenovirus pneumonia, bronchiolitis obliterans, risk factor, post-infectious bronchiolitis obliterans (PIBO)

## Abstract

**Objective:**

To preliminarily explore the risk factors for post-infectious bronchiolitis obliterans (PIBO) complicating adenovirus pneumonia (ADVP) in children through a meta-analysis.

**Methods:**

A systematic search was conducted on three English-language databases (PubMed, Web of Science and The National Library of Medicine) and two Chinese-language databases (China National Knowledge Infrastructure and the Wanfang Database) between database inception and 1 January 2023. Data analysis was conducted using Stata 15.1 software.

**Results:**

A total of 10 articles, reporting 14 risk factors, were included in the analysis, with 8 risk factors taken into consideration. Through the meta-analysis, 5 risk factors were identified for PIBO complicating ADVP in paediatric patients: hypoxaemia [odds ratio (OR) = 9.37, 95% CI: 4.22, 20.77, *p* < 0.001], persistent wheezing (OR = 4.65, 95% CI: 2.20, 9.82, *p *< 0.001), mechanical ventilation (OR = 3.87, 95% CI: 2.37, 6.33, *p *< 0.001), length of hospital stay (LoHS) (OR = 1.25, 95% CI: 1.09, 1.43, *p *< 0.001) and fever duration (OR = 1.08, 95% CI: 1.02, 1.14, *p =* 0.009).

**Conclusion:**

Existing evidence suggests that hypoxaemia, persistent wheezing, mechanical ventilation, LoHS and fever duration are risk factors for PIBO complicating ADVP in children. These findings underscore the need for enhanced assessment and management in clinical practice. This study may provide such a clinical prediction model from the identified 5 risk factors for PIBO and offer valuable insights for preventing bronchiolitis obliterans in children with ADVP.

## 1 Introduction

Adenovirus (ADV) is a common pathogen that causes acute respiratory infections in children, and its infections occur most often in children aged <5 years (1), with the prevalence of respiratory infections in children ranging from 5% to 10% (2); around one-third of ADV pneumonia (ADVP) cases can progress to severe disease (3). Bronchiolitis obliterans (BO) is a pathologic condition that usually involves fibrosis in small conducting airways, resulting in airway narrowing or complete obstruction (4). This condition is associated with various factors, including severe lower respiratory tract infections, haematopoietic stem cell transplantation, lung transplantation and connective tissue diseases (5, 6). Notably, the respiratory infections causing BO are also referred to as post-infectious BO (PIBO), representing a major type of paediatric BO (7). Adenovirus is one of the most common sources of infection leading to PIBO in children, accounting for 50% of PIBO cases (8). Previous studies have shown that up to 28% of paediatric patients who survive ADVP may go on to develop PIBO (9). Patients with PIBO typically develop diffuse lung disease and hypoxaemia due to a history of often severe lower respiratory tract infections (10), and many require intensive care and ventilation during treatment. After discharge from the hospital for the initial episode, a significant number of patients continue to experience wheezing and coughing (4); therefore, the occurrence of PIBO significantly affects the prognosis and long-term quality of life of children (11, 12). There are still no treatment guidelines for PIBO, and anti-inflammatory and symptomatic treatments are the mainstay, in addition to which children require regular follow-ups to monitor pulmonary function and lung computed tomography changes (4). However, the diagnosis of PIBO is usually based on a combination of clinical features, pulmonary function tests and radiologic findings, and there may be a significant lag between symptom presentation and diagnosis (13). Delayed diagnosis may lead to more serious complications, suggesting that early recognition can help to minimise further deterioration of the patient's condition. As a result, early identification of cases of ADVP in children at risk of developing PIBO and early initiation of interventions (14) are important to help children recover quickly and reduce complications.

In terms of paediatric patients, the essential prerequisite for implementing scientific interventions is the accurate identification of risk factors for PIBO following ADV infection. However, there is a lack of evidence-based studies on the risk factors for PIBO in children infected with ADV. Against this backdrop, the present study aims to employ a meta-analysis approach to explore the risk factors for PIBO after ADVP in children, thereby offering fresh insights for clinical practice.

## 2 Materials and methods

### 2.1 Literature retrieval strategy

Following the guidelines of the Preferred Reporting Items for Systematic Reviews and Meta-Analyses, a systematic search was conducted in three English-language databases, namely PubMed, Web of Science and The National Library of Medicine, and two Chinese-language databases, namely China National Knowledge Infrastructure and the Wanfang Database. The search period extended from the establishment of these databases until 1 January 2023. A combination of controlled vocabulary and free-text terms were used during the search. The search strategy for keywords was as follows: (Adenoviridae OR Adenovirus) AND (Infants OR Children) AND (risk factors OR Influencing factors) AND (pneumonia Infection OR Bronchiolitis Obliterans OR Cryptogenic Bronchiolitis Obliterans). These searches identified the published literature that discussed the risk factors for BO in children after ADV infection.

### 2.2 Inclusion and exclusion criteria

The inclusion criteria were as follows: (1) study types: case-control studies or cohort studies; (2) study participants: children who developed BO after ADV infection; and (3) risk factors: all risk/predictive factors associated with BO following the occurrence of ADVP.

The exclusion criteria were as follows: (1) BO not caused by infection; (2) duplicate publications; (3) literature lacking combined effect sizes for statistical analysis; (4) studies assessed as low-quality literature, and/or (5) lack of clear diagnostic criteria.

### 2.3 Literature screening and data extraction

Literature screening was undertaken by two researchers independently. Initial screening was based on titles and abstracts, followed by a second screening of full texts according to the inclusion and exclusion criteria. In cases of disagreement, the opinion of a third researcher was sought, and discussions were held to reach a consensus. Following literature screening, data extraction was conducted by two researchers independently. Extracted information included the first author, publication year, study region, study type, sample size and all included risk factors.

### 2.4 Literature quality assessment

The Newcastle–Ottawa Scale (NOS) was employed to assess the quality of the included cohort and case-control studies. The evaluation covered three aspects: selection, comparability and exposure or outcomes. The scale has a total score of 9 points, with 1 point awarded for meeting each scoring criterion. Studies with a score of <5 points were considered low-quality, while those with a score of ≥5 were classified as high-quality. Studies with an NOS score of <5 were not included in the meta-analysis (15).

### 2.5 Statistical methods

Meta-analysis was performed using the software Stata 15.1. For continuous data, the mean difference was used as the effect measure if the continuous data was normally distributed, while for categorical data, the odds ratio (OR) was employed. Point estimates and 95% CI were used to represent each effect size. Heterogeneity was assessed using the *I*^2^ test, with *I*^2^ ≤ 50% or *p* ≥ 0.1 indicating homogeneity among the included studies. In such cases, the Mantel–Haenszel fixed-effects meta-analysis methods were applied. If *I*^2^ ≥ 50% or *p* ≤ 0.1, which suggests a certain degree of heterogeneity among the included studies, the DerSimonian and Laird random-effects meta-analysis method was used. The significance level for the meta-analysis was set at *α* = 0.05.

## 3 Results

### 3.1 Literature retrieval results

**Figure 1:**
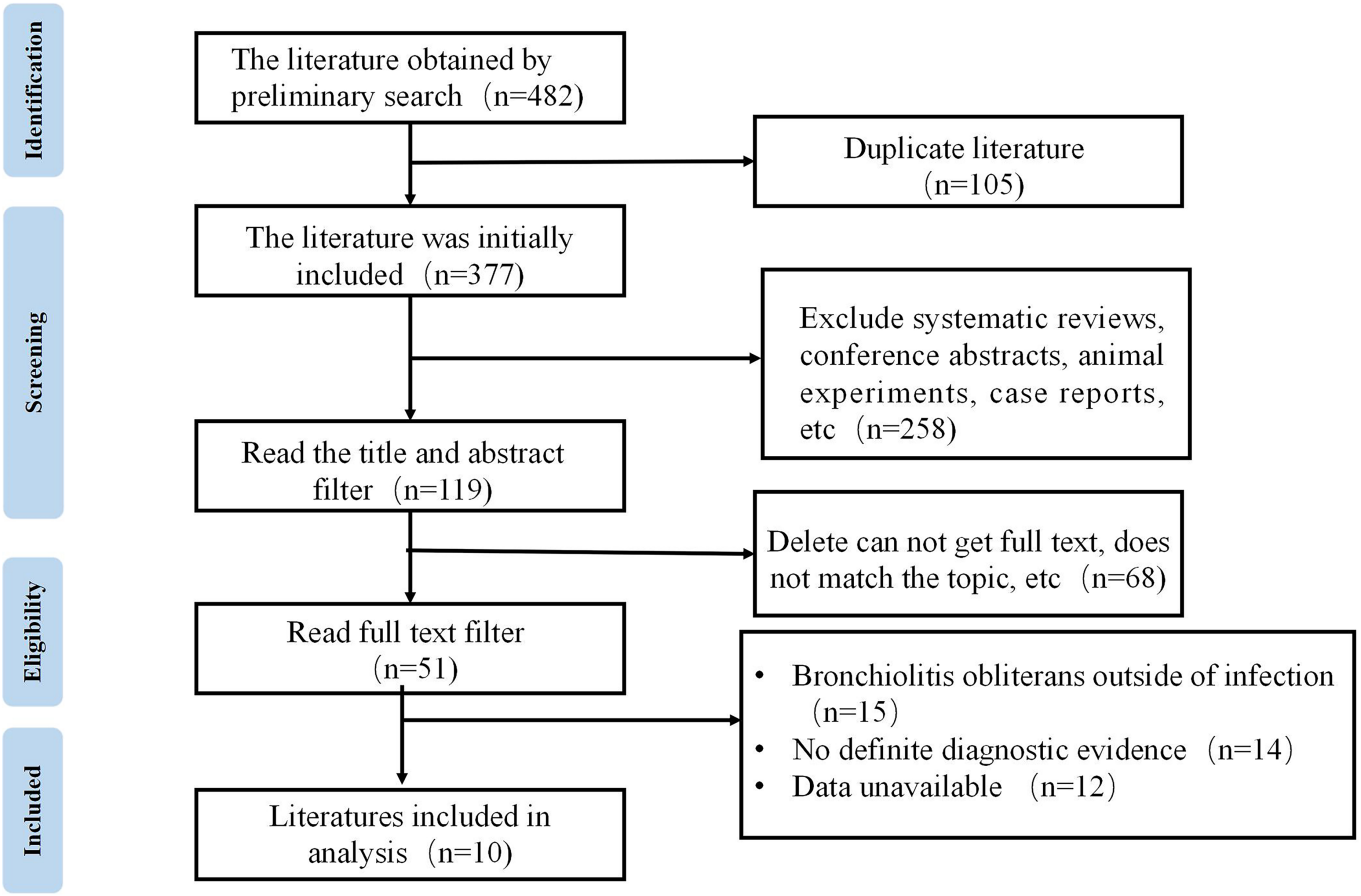
Literature screening process.

A total of 482 articles were retrieved through the database searches. Among these articles, 105 were duplicates, and 258 were systematic reviews and case reports—these studies were excluded. Following thorough full-text reading, studies lacking clear diagnostic criteria or investigating occlusive bronchiolitis of causes other than infection were excluded according to the given criteria. Ultimately, 10 articles were included for meta-analysis (9, 16–23). See Figure 1 for the literature screening process.

### 3.2 Fundamental characteristics of included literature and quality assessment results

The 10 included articles, published between 2009 and 2023, consisted of six retrospective studies, one cohort study and three case-control studies. A total of 482 patients with PIBO were included. The quality assessment scores for the included retrospective and prospective cohort studies ranged from 6 to 8, indicating medium to high quality according to the inclusion criteria. See Table 1 for the fundamental characteristics and quality assessment results of the included literature.

**Table 1:** Fundamental characteristics of included literature and quality assessment results.

Included Literature	Publication year	Study type	Sample size	Age (months)	Risk factors	Literature quality score
Murtagh et al. (9)	2009	Retrospective study	117	10.7 ± 9.2	4, 6	6
Zhong et al. (16)	2020	Case-control study	34	15.1 ± 7.2	2, 4, 6, 8, 9, 10, 11, 13	8
Wu et al. (17)	2016	Retrospective study	14	15.5 ± 16.5	4, 6, 7, 10, 12	7
Dai et al. (18)	2020	Retrospective study	37	12.0 ± 7.1	3, 12, 14	7
Yu et al. (19)	2021	Case-control study	20	16.5 ± 10.56	2, 3, 4, 6, 10, 12, 14	7
Peng et al. (20)	2023	Cohort study	46	12 ± 9.63	6, 8, 10	8
Lee and Young Lee (21)	2020	Retrospective study	18	–	1, 4, 11, 13	7
Lan	2021	Retrospective study	69	25 ± 23.0	4, 5, 6, 7, 10, 11, 13, 14	7
Li et al. (22)	2020	Retrospective study	98	18.28 ± 15.17	3, 4, 6, 7, 10, 11, 14	7
Li et al. (23)	2021	Case-control study	29	27.7 ± 19.5	3, 4, 11, 13, 14	8

1 = respiratory failure; 2 = breathing difficulty; 3 = persistent wheezing; 4 = length of hospital stay; 5 = ICU admission; 6 = invasive mechanical ventilation; 7 = use of corticosteroids; 8 = extrapulmonary complications; 9 = anemia; 10 = fever duration; 11 = lactate dehydrogenase; 12 = hypoxia; 13 = concurrent bacterial infection; 14 = age.

### 3.3 Meta-analysis results

Based on the contents of the 10 included articles, 14 factors were identified as risk factors for the development of BO in children following ADVP. A meta-analysis was conducted for factors present in >3 articles, and a total of 8 risk factors were includedin the analysis. The meta-analysis results revealed that persistent wheezing, length of hospital stay (LoHS), mechanical ventilation, fever duration, lactate dehydrogenase (LDH) and hypoxaemia were significant risk factors for BO in children with ADVP (see Table 2). The definition of LoHS refers to the duration of a single hospitalization, not the cumulative duration of hospitalization.

**Table 2:** Significant risk factors for BO in children with adenovirus pneumonia.

Risk factors	Number of studies	Heterogeneity test		Model	Pooled results	
		*I*^2^ (%)	*P*		OR (95%CI)	*P*
Persistent wheezing	4	13.6	0.325	Fixed-effects model	4.65 (2.20,9.82)	<0.001*
Length of hospital stay	8	95.8	<0.001	Random-effects model	1.25 (1.09,1.43)	<0.001*
Mechanical ventilation	7	62.7	0.013	Random-effects model	3.87 (2.37,6.33)	<0.001*
Fever duration	6	77.7	0.001	Random-effects model	1.08 (1.02,1.14)	0.009*
Lactate dehydrogenase	5	32.7	0.204	Fixed-effects model	1.00 (1.00,1.00)	<0.001*
Hypoxemia	3	0.0	0.385	Fixed-effects model	9.37 (4.22,20.77)	<0.001*
Concurrent bacterial infection	4	86.8	<0.001	Random-effects model	1.21 (0.28,5.12)	0.800
Age	5	76.8	0.002	Random-effects model	1.08 (0.99,1.18)	0.076

* *P *< 0.05.

#### 3.3.1 Hypoxaemia

**Figure 2:**
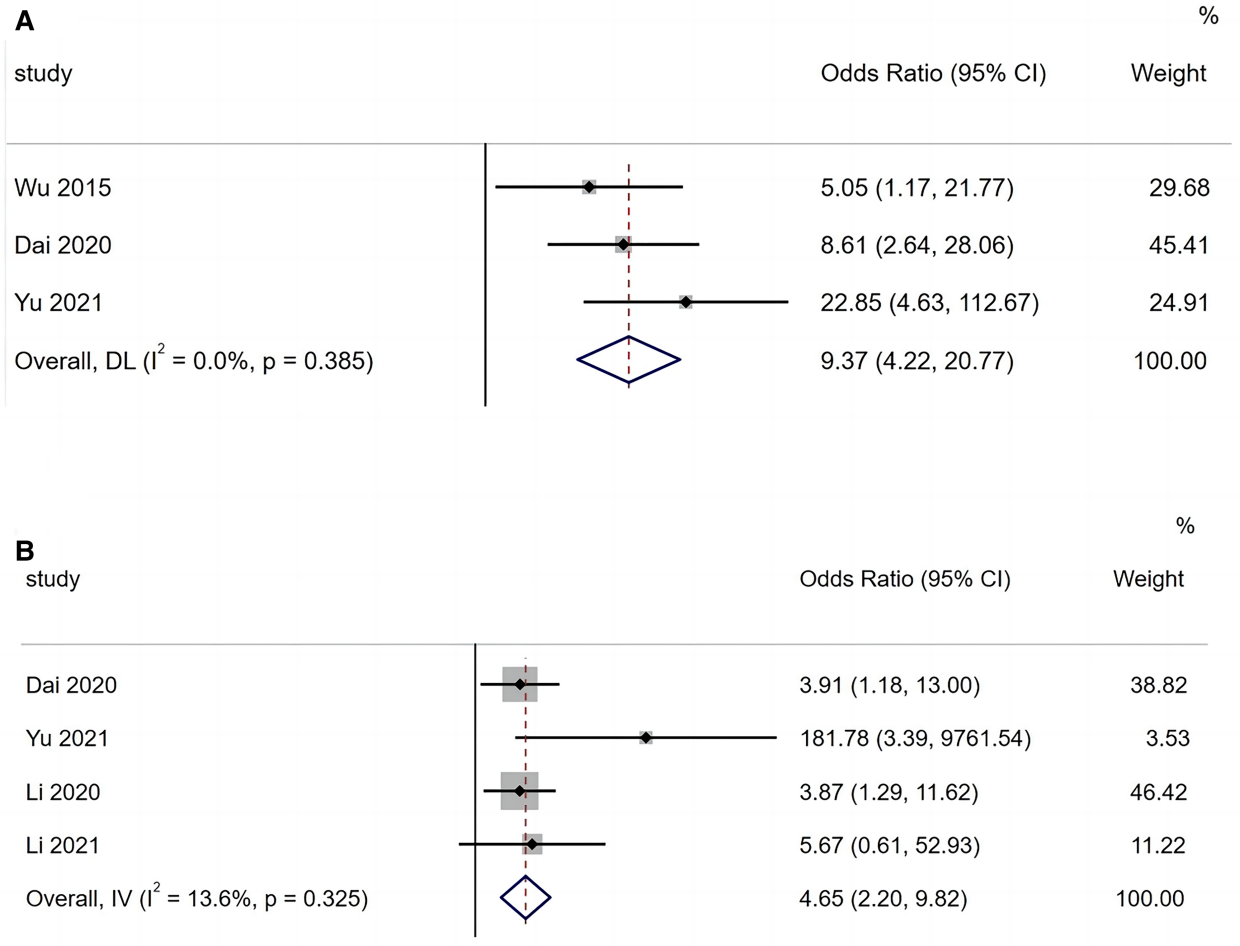
(**A**) Forest plot of hypoxemia and bronchiolitis obliterans in children with adenovirus pneumonia; (**B**) forest plot of persistent wheezing and bronchiolitis obliterans in children with adenovirus pneumonia.

A total of three articles reported that hypoxaemia is a risk factor for BO in children with ADVP. As shown in Figure 2A, the studies are highly homogeneous (*I*^2^ = 0.0%). Based on a fixed-effects model, the results of the meta-analysis indicated that hypoxaemia is a significant risk factor for BO in children with ADVP (OR = 9.37, 95% CI: 4.22, 20.77, *p *< 0.001).

#### 3.3.2 Persistent wheezing

Of the 10 included articles, four reported persistent wheezing as a risk factor for BO in children with ADVP. As shown in Figure 2B, there is low heterogeneity among these studies (*I*^2^ = 13.6%). A fixed-effects model was applied to the meta-analysis, and the results identified persistent wheezing as a significant risk factor for BO in children with ADVP (OR = 4.65, 95% CI: 2.20, 9.82, *p *< 0.001).

#### 3.3.3 Mechanical ventilation

**Figure 3:**
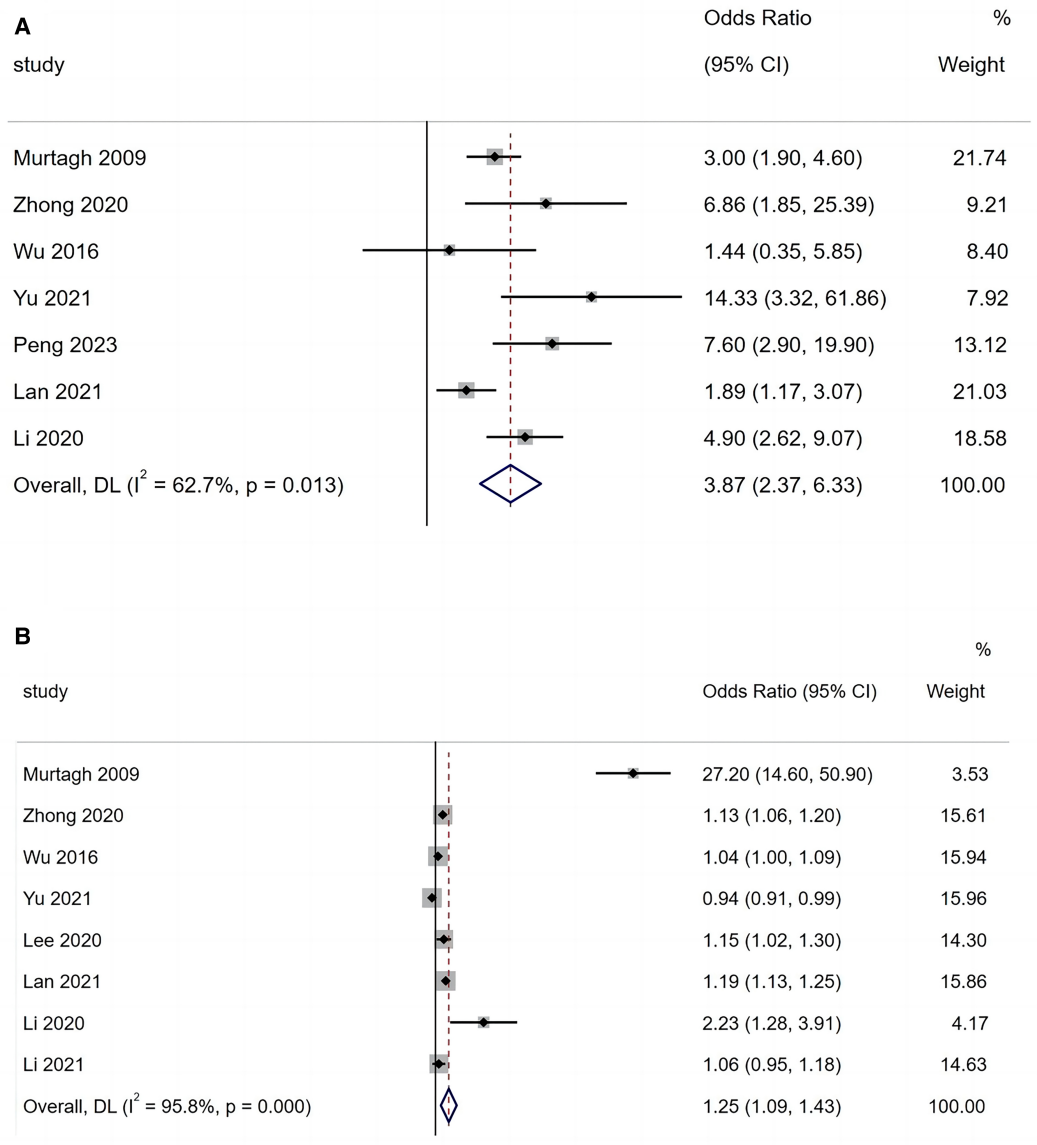
(**A**) Forest plot of mechanical ventilation and bronchiolitis obliterans in children with adenovirus pneumonia; (**B**) forest plot of length of hospital stay and bronchiolitis obliterans in children with adenovirus pneumonia.

A total of seven articles reported that mechanical ventilation is a risk factor for BO in children with ADVP. As shown in Figure 3A, there was heterogeneity among the studies (*I*^2^ = 62.7%). A random-effects model was used for the meta-analysis, and the results indicated that mechanical ventilation is a significant risk factor for BO in children with ADVP (OR = 3.87, 95% CI: 2.37, 6.33, *p *< 0.001).

#### 3.3.4 Length of hospital stay

A total of eight articles reported that LoHS is a risk factor for BO in children with ADVP. As shown in Figure 3B, there was high heterogeneity among these studies (*I*^2^ = 95.8%). In this case, a random-effects model was employed in the meta-analysis, and the results indicated that LoHS is a significant risk factor for BO in children with ADVP (OR = 1.25, 95% CI: 1.09, 1.43, *p *< 0.001).

#### 3.3.5 Fever duration

**Figure 4:**
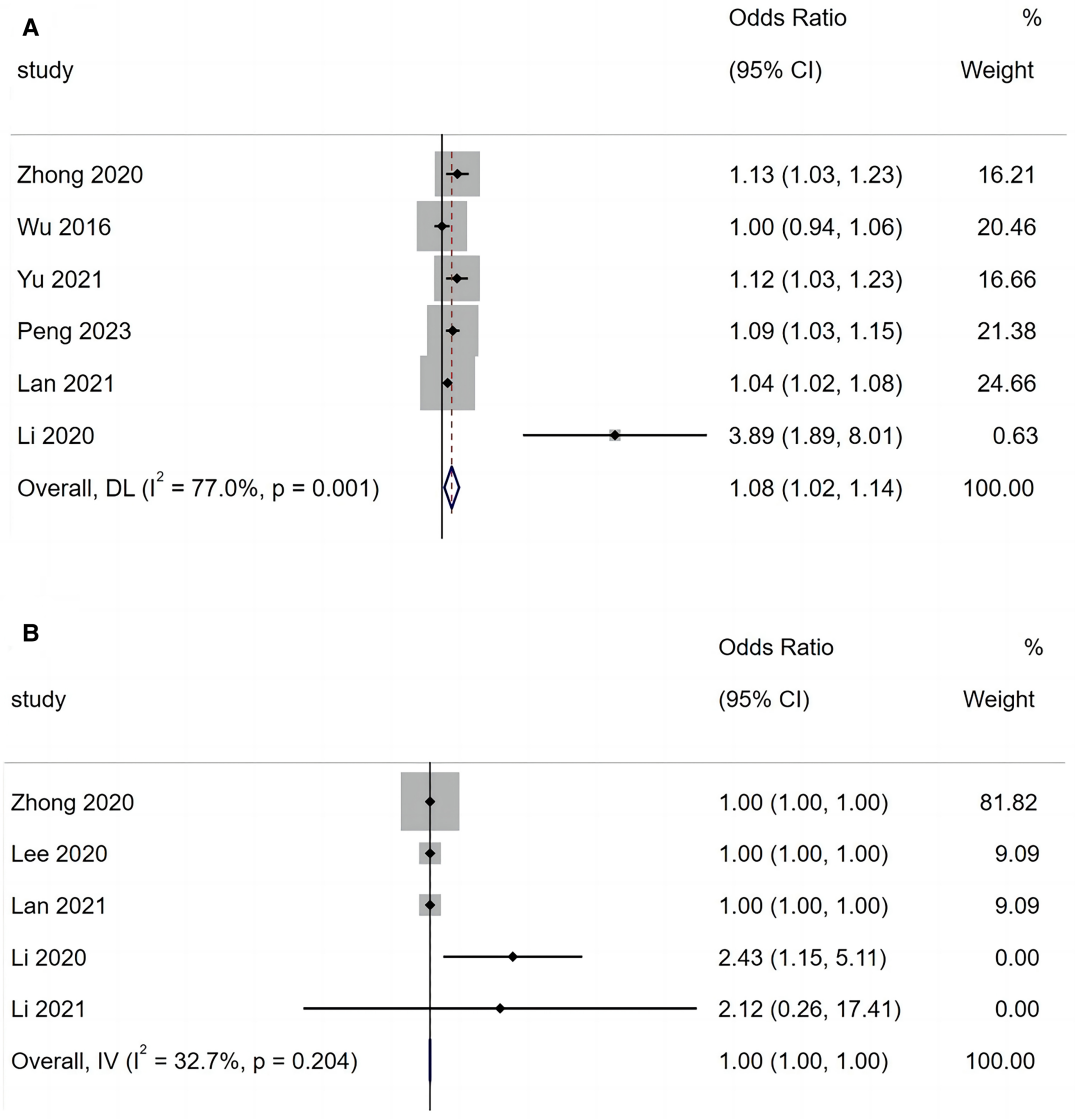
(**A**) Forest plot of fever duration and bronchiolitis obliterans in children with adenovirus pneumonia; (**B**) forest plot of lactate dehydrogenase and bronchiolitis obliterans in children with adenovirus pneumonia.

A total of six articles reported that fever duration is a risk factor for BO in children with ADVP. As shown in Figure 4A, there was heterogeneity among the studies (*I*^2^ = 77.7%). Using a random-effects model for meta-analysis, the results indicated that fever duration is a significant risk factor for BO in children with ADVP (OR = 1.08, 95% CI: 1.02, 1.14, *p =* 0.009).

#### 3.3.6 Lactate dehydrogenase

A total of five articles reported that LDH is a risk factor for BO in children with ADVP. As shown in Figure 4B, there was low heterogeneity among the studies (*I*^2^ = 32.7%). A fixed-effects model was adopted to conduct a meta-analysis, and the results revealed that LDH is a significant risk factor for BO in children with ADVP (OR = 1.00, 95% CI: 1.00, 1.00, *p *< 0.001).

#### 3.3.7 Concurrent bacterial infection

**Figure 5:**
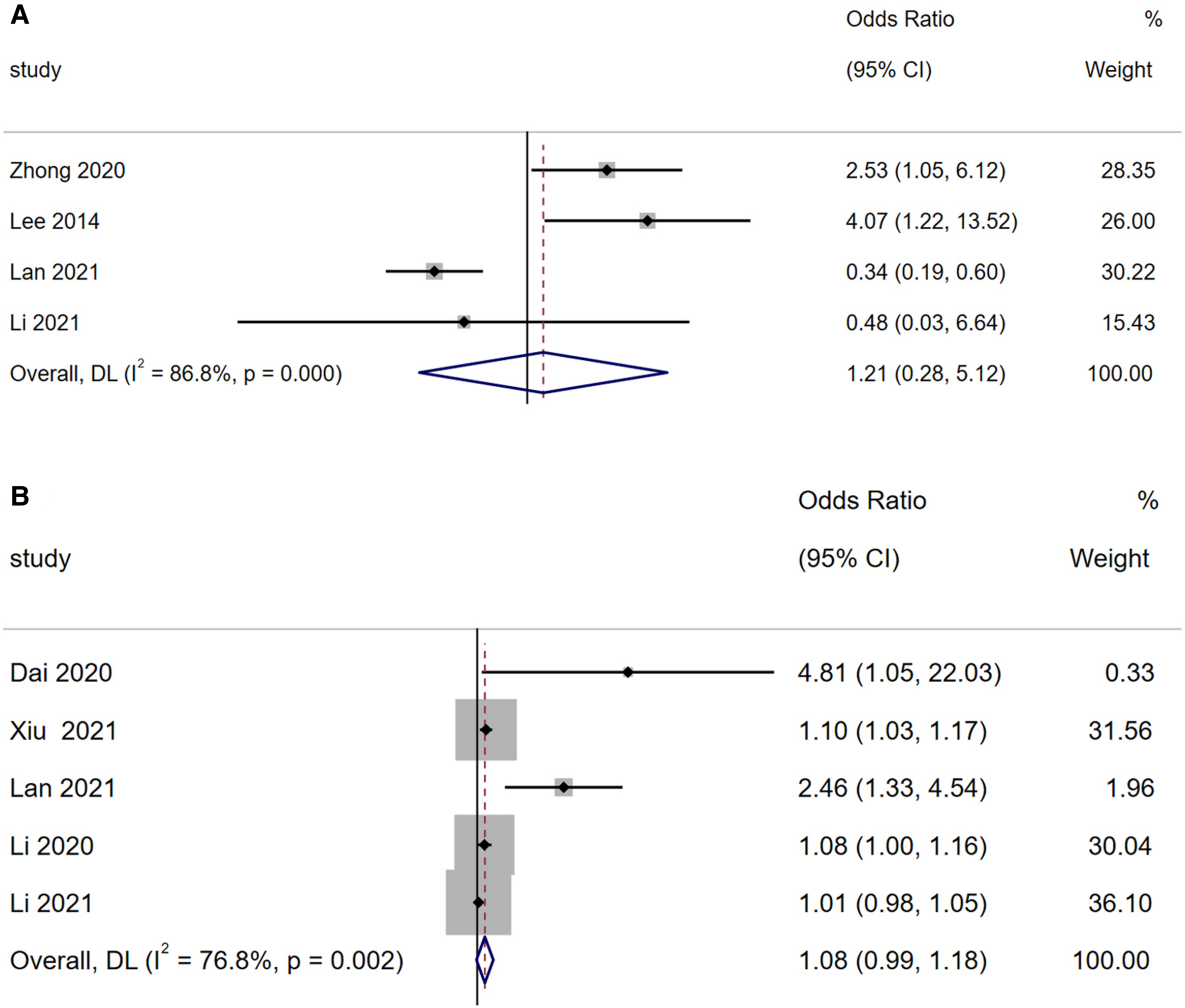
(**A**) Forest plot of concurrent bacterial infection and bronchiolitis obliterans in children with adenovirus pneumonia; (**B**) forest plot of age and bronchiolitis obliterans in children with adenovirus pneumonia.

A total of four articles reported that concurrent bacterial infection is a risk factor for BO in children with ADVP. As shown in Figure 5A, there was high heterogeneity among the studies (*I*^2^ = 86.8%). With a random-effects model, the meta-analysis suggested that concurrent bacterial infection is not a significant risk factor for BO in children with ADVP (OR = 1.21, 95% CI: 0.28, 5.12, *p =* 0.800).

#### 3.3.8 Age

A total of five articles reported that age is a risk factor for BO in children with ADVP. As shown in Figure 5B, there was high heterogeneity among the studies (*I*^2^ = 76.8%). Using a random-effects model for meta-analysis, the results indicated that age is not a significant risk factor for BO in children with ADVP (OR = 1.08, 95% CI: 0.99, 1.18, *p =* 0.076).

## 4 Discussion

It is crucial to identify the risk of PIBO and initiate early intervention for children with ADVP. At present, there is a lack of evidence-based risk factors for PIBO following ADV infection in paediatric patients. Therefore, a meta-analysis approach was adopted in this study to investigate and analyse the risk factors for PIBO in children with ADVP. Based on the meta-analysis of 10 studies, the results revealed that hypoxaemia, persistent wheezing, mechanical ventilation, LoHS and duration of fever were risk factors for the development of BO in children with ADVP. In contrast, the results regarding LDH, bacterial infection and age as risk factors lacked statistical significance.

The meta-analysis identified hypoxaemia as a risk factor for BO in children with ADVP. Wu et al. (17) noted that, as a newly discovered independent predictor for BO in paediatric ADV infection cases, hypoxaemia appears to be more sensitive than other risk factors (17). However, the mechanism by which hypoxaemia influences BO remains unclear, warranting further exploration through large-scale, multicentre studies.

Consistent with previous research findings, Chen et al. reported (24) that wheezing during the acute phase of ADVP is a risk factor for PIBO in paediatric patients. Persistent wheezing reflects adenoviral damage to the small airways and inflammatory processes. In addition, persistent wheezing in young children increases the incidence of childhood asthma, so physicians should pay special attention to wheezing in children and intervene early with medications if the patient's condition worsens.

In addition, mechanical ventilation and LoHS have been identified as risk factors for PIBO in children. The rapid progression of ADVP, particularly in severe cases, often requires respiratory support and, in some cases, invasive mechanical ventilation. The use of mechanical ventilation implies an increased degree of lung pathology and inevitably causes mechanical damage to lung tissue, which is more likely to promote the development of BO. Related studies (16) suggest that children with ADVP who develop BO tend to have a longer hospital stay, indicating either a more severe condition or poorer treatment outcomes. In other words, the occurrence of BO is potentially associated with the severity of ADVP. Additionally, our study results identified fever duration as a risk factor for BO in children with ADVP, consistent with the findings of several previous studies (9, 25). Prolonged fever may indicate the persistence of inflammatory symptoms in children, activation of the immune system by inflammatory mediators and damage to multiple organ systems. Persistent hyperthermia in a state of elevated inflammatory response may ultimately lead to a vicious cycle resulting in the development of PIBO. Li Peng et al. (20) found that the percentage of neutrophils was an independent risk factor for the development of PIBO in children with ADVP, which confirms the notion that the condition is exacerbated by inflammation. In previous studies, persistent fever could also be considered a sign of a high inflammatory response (26).

In this study, the results indicate that LDH, age and concurrent bacterial infection are not statistically significant as risk factors for BO in children with ADVP. However, researchers in other studies investigated eight severely ill paediatric patients with ADVP requiring extracorporeal membrane oxygenation support and discovered a notable increase in LDH, reaching 14,585 (U/L) (6,348–32,448), compared with non-severe cases (408.0 (U/L) [282.0–639.0]) (25). Combined bacterial infections may not affect the occurrence of BO after adenoviral infection, and Liu et al. (27) also found no statistically significant differences in clinical features and laboratory findings between patients with single ADV infection and mixed bacterial infection. However, it is worth noting that bacterial infections that produce inflammation can also produce symptoms such as fever, which, in turn, increases the risk of BO. Furthermore, other studies have suggested that younger age is indeed a risk factor for BO following ADVP. Bronchiolitis obliterans is more likely to occur in children aged between 6 months and 2 years (28). A retrospective case-control study in Singapore by Raikumar et al. (29) assessed 85 children hospitalised for ADVP. The study found that infants aged <2 years were more susceptible to developing severe conditions, leading to serious complications, such as BO and bronchiectasis. This may be explained by the fact that >90% of infants acquire antibodies against human ADV (HADV) through the placenta at birth. These antibodies protect infants aged <6 months from HADV infection. Reportedly, only 14% of infants tested positive for complement-fixing antibodies to HADV types at 6 months of age. The decrease in these antibodies corresponds to a significant increase in the incidence of ADVP in children aged >6 months, particularly in those with compromised immune function or chronic underlying conditions. Consequently, the risk of developing BO is elevated in this population after ADVP (30). In short, age remains a crucial factor that requires special attention in paediatric cases of ADVP.

In this study, the meta-analysis included 10 articles and was strictly implemented according to the established procedures. The extraction of single- and multiple-factor regression results from the articles has, to a certain extent, mitigated the influence of uncertainties, thereby enhancing the reliability of the study results and providing a basis for the prevention of PIBO in children with ADVP. Despite this, there are limitations in this meta-analysis. First, the included literature comprised mostly retrospective studies. Second, some study results have incomplete parameters, and the number of risk factors included in the literature analysis was relatively small. Therefore, multicentre cohort studies with a large sample size are needed to validate the findings presented in this study.

## 5 Conclusion

This study was based on a meta-analysis of 10 studies, which found that hypoxaemia, persistent wheezing, mechanical ventilation, LoSH and duration of fever were risk factors for the development of BO in children with ADVP.

## Data Availability

The original contributions presented in the study are included in the article/Supplementary Material, further inquiries can be directed to the corresponding author.
